# Non-linear relationship between platelet count and 28-day mortality in critically ill patients with infective endocarditis: a retrospective cohort study from MIMIC IV database

**DOI:** 10.3389/fcvm.2024.1458238

**Published:** 2024-11-29

**Authors:** Yingxiu Huang, Ting Ao, Peng Zhen, Ming Hu

**Affiliations:** Department of Infectious Disease, Beijing Luhe Hospital, Capital Medical University, Beijing, China

**Keywords:** platelet count, infective endocarditis, critically ill patients, nonlinear relationship, mortality, MIMIC-IV

## Abstract

**Background:**

The relationship between platelet count and 28-day mortality in critically ill patients with infective endocarditis (IE) is currently not well established.

**Objective:**

This study aims to investigate the impact of platelet count on 28-day mortality in critically ill patients with infective endocarditis.

**Methods:**

A retrospective cohort study was conducted involving 450 participants diagnosed with infective endocarditis and admitted to intensive care units (ICU). Vital signs, laboratory parameters and comorbidity were collected for all participants to analyze the association between platelet count and 28-day mortality. In order to assess the independent association between platelet count and 28-day mortality, we employed multivariable cox hazard regression analyses and smooth curve fitting. A further analysis was conducted using a two-piecewise linear regression model to examine the nonlinear association between platelet count and in-hospital mortality.

**Results:**

A total of 450 critically ill patients with infective endocarditis were included in the study. The mean age was 57.4 years, and 64.2% were male. The overall 28-day mortality rate was 20%. A non-linear relationship was observed between platelet count and 28-day mortality. Two different slopes were identified, with correlations between platelet count and 28-day mortality in patients with IE differing significantly below and above the inflection point, which was approximately 141 K/µl. On the left side of the inflection point, the hazard ratio was 0.990 (hazard ratio: 0.990, 95% confidence interval: 0.982–0.997, *p* = 0.006). However, on the right side of the inflection point, the hazard ratio increased marginally to 1.0004 (HR: 1.0004, 95% CI: 0.997–1.004, *p* = 0.825). Notably, the association lacked statistical significance on the right side of the inflection point.

**Conclusion:**

A nonlinear association between platelet count and 28-day mortality was observed in critically ill patients with infective endocarditis. The optimal platelet count associated with the lowest risk of 28-day mortality was above 141 k/µl.

## Introduction

Infective endocarditis (IE), typically caused by bacterial or fungal infections of the inner lining of the heart chambers and heart valves, can lead to serious complications such as heart damage, stroke, and organ failure (1). Symptoms may include fever, chills, fatigue, weakness, and changes in heart rhythm. Treatment usually involves a combination of antibiotics and, in some cases, surgery to repair or replace damaged heart valves. The incidence of IE is 3–10 per 100,000 people (2–4). Despite advancements in diagnostic tools and therapeutic measures, the in-hospital mortality rate due to IE remains at approximately 25% (5), leading to a high economic burden with average hospitalization costs ranging from $37,000 to $55,000 per patient (6). The prognosis of patients with infective endocarditis is often influenced by multiple factors, including the severity of the infection and the timeliness of early diagnosis and treatment. Various factors, including individual patient characteristics, cardiac and non-cardiac comorbidities, infecting microbial species, and echocardiographic disease status, contribute to the poor prognosis of IE (3, 7). Early diagnosis is critical to improving patient outcomes and reducing mortality associated with IE (8). However, research on preventive factors for infective endocarditis is limited, presenting a challenge. In clinical practice, it is crucial to identify reliable predictive markers to assess disease progression and prognosis, which can guide treatment decisions.

Platelets are small bioactive particles generated by the cytoplasmic lysis of bone marrow megakaryocytes (9). Platelets are increasingly acknowledged for their essential role in inflammation and immune responses (10). Thrombocytopenia, characterized by low platelet counts, is a prevalent condition in critically ill patients both upon admission to and during their ICU stay. Previous research has consistently indicated a correlation between thrombocytopenia and lower platelet counts and increased mortality rates in different situation, such as sepsis (11), covid-19 (12, 13), stroke (14), acute respiratory failure (15), acute myocardial infarction (16).

However, the association between admission platelet count levels and the mortality risk of IE remains unclear. Therefore, the primary aim of this study is to investigate the association between admission platelet count and the risk of 28-day mortality in patients diagnosed with IE. We hypothesized that a lower platelet count is associated with higher mortality in critically ill patients with IE.

## Method

### Database

The Medical Information Mart for Intensive Care IV (MIMIC IV) database v2.2 (https://mimic.mit.edu/) provided the data used in this study (17). It includes details on 73,181 hospitalizations of critically ill patients who were admitted to Boston's Beth Israel Deaconess Medical Center between 2008 and 2019 (18). Numerous data elements are included in the database, including survival statue, vital signs, laboratory indicators, illness names, and treatment plans. Because of the MIMIC database's abundance of high-quality data and its other benefits, an increasing number of academics are using it to conduct research (19–21). Two of the authors, Yingxiu Huang, and Ting Ao were given access to the database following successful completion of an online course and test (Certificate ID: 56513391 Yingxiu Huang, Certificate ID: 58844105 Ting Ao). Since the database protects patients' privacy, informed permission was not required. The present cohort study was in compliance with the Strengthening the Reporting of Observational Studies in Epidemiology (STROBE) (22).

The retrospective cohort study utilized data from MIMIC IV version 2.2. We included patients diagnosed with infective endocarditis who were admitted to the ICU for the first time in our study. Our primary endpoint was the evaluation of 28-day mortality. We gathered information on patient demographics: sex, age, race; vital signs: heart rate, mean blood pressure (MBP), respiratory rate, spo2; comorbidities: congestive heart failure, renal disease, diabetes, chronic pulmonary disease, and sepsis; severity of illness score: Charlson Comorbidity Index, laboratory results: platelet count, white blood cell (WBC), creatinine, sodium, potassium, bicarbonate, lactate, glucose, and blood culture result (Staphylococcus spp., Streptococcus spp., Enterococcus spp., HACEK organisms, double infection, culture-negative). The severity of illness was evaluated using the Charlson Comorbidity Index (CCI). The comorbidities assessed comprised congestive heart failure, renal disease, diabetes, chronic pulmonary disease, and sepsis. Sepsis was defined according to the criteria outlined in Sepsis 3.0 (23).

### Study population

#### Criteria

First, we included patients who had been diagnosed with infective endocarditis during their hospital admission from 2008 to 2019, and the diagnostic criteria were based on the International Classification of Diseases(ICD), ICD9 and ICD10(code = 03642, 07422, 0932X, 09884, 11281, 11504, 11514, 11594, 3911, 421X, 4249X, A3282, A3951, A5203, B3321, B376, I011, I38, I33, I330, I339, I39, M3211). Second, only the first ICU admission record was selected. Finally, we limited our analysis to individuals whose platelet count were obtained within 24 h of their ICU admission. If several measurements were acquired within the first 24 h, the lowest platelet count was selected and retained for analysis.

#### Covariates

Relevant information was extracted from the MIMIC-IV database using a structured query language were stored in PostgreSQL. The extracted data included patient demographics such as age and sex, race; laboratory indicators such as WBC, hemoglobin, creatinine, lactate, and glucose on the first day of ICU admission, and comorbidity.

### Statistical analysis

Patients were classified into four groups based on the quartile distribution of platelet count. The characteristics were summed together using descriptive statistics. Continuous variables were expressed as mean ± standard deviation (SD) or median [interquartile range (IQR)], while categorical variables were presented as proportions (percentages). The means of continuous variables were shown with their standard deviations. Counts and percentages were used to display categorical variables. For the analysis of baseline characteristics, data were compared using the Mann–Whitney test for continuous variables and the chi-square test for categorical variables.

To assess the independent relationship between platelet count and 28-day mortality, we utilized multivariable Cox hazards regression models to determine hazard ratios (HRs) and 95% confidence intervals (CIs) for the association between platelet quartiles and mortality risk. Adjusted models, incorporating various variables, were constructed using an extended Cox model, with the lowest quartile of platelet count serving as the reference group. Model 1 was unadjusted. Model 2 adjusted for age, gender, race. Model 3 further adjusted for comorbidities congestive heart failure, renal disease, chronic pulmonary disease, and sepsis. Model 4: Adjust for age, sex, race, sepsis, congestive heart failure, chronic pulmonary disease, renal disease, creatinine, glucose, and CCI.

In addition, we employed smooth curve fitting to assess the presence of a dose-response relationship between 28-day mortality and platelet count. To compare the two-piecewise linear model with the one-line linear regression model, a likelihood ratio test was performed. Also, log-rank analysis was used to compare the survival curves that were produced using Kaplan-Meier analysis. Stratified and interaction analyses were conducted to evaluate the consistency of the association between platelet count and 28-day mortality across various subgroups, including age (<65 or ≥65 years), sex, race, diabetes, congestive heart failure, renal disease, CCI (<6 or ≥6), and sepsis. Additionally, we conducted a sensitivity analysis using patients with platelet counts above 141,000/μl (considered normal) as the reference group, while subdividing those with platelet counts below 141,000/μl into three categories: mild thrombocytopenia (101–140 K/μl), moderate thrombocytopenia (50–100 K/μl), and severe thrombocytopenia (<50 K/μl).

All of the studies employed the statistical software packages R 3.3.2 (http://www.R-project.org, The R Foundation) and Free Statistics software versions 1.9.2 (24). A statistically significant result was defined as *p* < 0.05 after a two-tailed test.

## Results

### Participants characteristics

**Figure 1:**
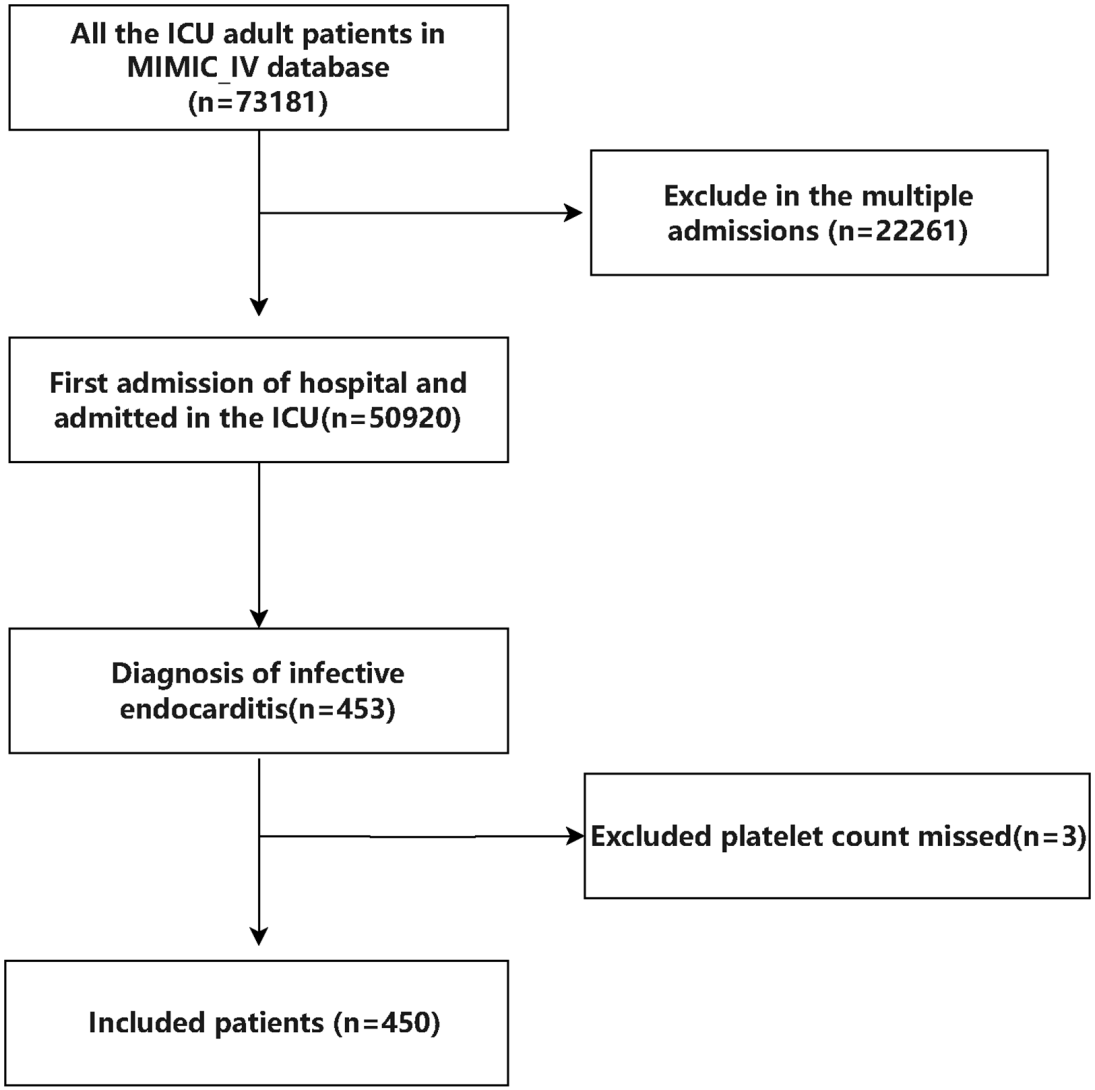
Flow chart of the study.

Initially, there were 453 patients with infective endocarditis enrolled to the ICU for the first admission in the MIMIC IV database. Following stringent screening in accordance with the inclusion and exclusion criteria, 450 patients in total were enrolled. A total of 450 patients, with a median age of 57 years and 64% males, were included in the final analysis after 3 patients were excluded owing to missing platelet count data (see Figure 1 for details). The 28-day mortality rate for the patients that were included was 20%. All individuals' baseline characteristics are shown in Table 1.

**Table 1:** Baseline characteristics of participants.

Variables		Platelet count (k/µl)				
	Total (*n* = 450)	Q1(≤99) (*n* = 111)	Q2 (100–156) (*n* = 113)	Q3 (157–234) (*n* = 111)	Q4(≥235) (*n* = 115)	*P* value
Sex, male, *n* (%)	289 (64.2)	69 (62.2)	79 (69.9)	75 (67.6)	66 (57.4)	0.197
Age, (years)	57.4 ± 18.0	54.3 ± 19.2	59.7 ± 17.3	56.8 ± 17.0	58.5 ± 18.3	0.124
Race, white, *n* (%)	308 (68.4)	75 (67.6)	74 (65.5)	83 (74.8)	76 (66.1)	0.415
heart rate, (bpm)	108.4 ± 21.7	116.4 ± 23.8	107.8 ± 22.1	103.4 ± 18.7	106.1 ± 19.7	<0.001
MBP (mmHg)	75.5 ± 10.4	76.0 ± 11.1	75.2 ± 10.1	76.7 ± 9.7	74.2 ± 10.6	0.299
respiratory rate (bpm)	31.2 ± 7.7	33.6 ± 8.7	30.6 ± 7.3	29.2 ± 7.0	31.5 ± 7.0	<0.001
spo2(%)	91.5 ± 6.8	90.1 ± 10.9	92.1 ± 4.9	92.5 ± 4.2	91.3 ± 5.0	0.057
glucose (ug/dl)	126.9 (110.1, 153.6)	125.9 (112.3, 159.5)	125.7 (111.7, 152.0)	124.0 (106.6, 143.6)	130.4 (113.5, 155.8)	0.395
WBC, (k/µl)	14.9 (10.6, 21.9)	14.5 (10.2, 22.7)	15.3 (11.4, 21.3)	14.2 (9.8, 21.0)	15.1 (11.2, 22.1)	0.735
Creatinine (mg/dl)	1.1 (0.8, 1.8)	1.3 (0.9, 2.0)	1.2 (0.8, 2.0)	1.0 (0.8, 1.7)	1.0 (0.8, 1.5)	0.021
Sodium (mEq/L)	138.4 ± 5.0	138.6 ± 5.6	139.0 ± 4.9	138.6 ± 4.7	137.4 ± 4.7	0.07
Potassium (mEq/L)	4.5 ± 0.7	4.4 ± 0.9	4.6 ± 0.7	4.6 ± 0.8	4.5 ± 0.6	0.131
Bicarbonate (mEq/L)	24.1 ± 4.2	22.3 ± 3.5	23.4 ± 4.0	24.9 ± 4.0	25.6 ± 4.4	<0.001
PT (s)	18.7 ± 9.5	18.3 ± 6.4	19.0 ± 8.2	18.2 ± 10.1	19.1 ± 12.4	0.857
Lactate (mmol/L)	2.7 ± 2.3	3.2 ± 3.0	2.9 ± 2.6	2.3 ± 1.5	2.2 ± 1.4	0.001
PH	7.3 ± 0.1	7.3 ± 0.1	7.3 ± 0.1	7.3 ± 0.1	7.3 ± 0.1	0.061
Diabetes, *n* (%)	121 (26.9)	21 (18.9)	35 (31)	29 (26.1)	36 (31.3)	0.126
Sepsis, *n* (%)	319 (70.9)	100 (90.1)	84 (74.3)	69 (62.2)	66 (57.4)	<0.001
Congestive heart failure, *n* (%)	193 (42.9)	36 (32.4)	57 (50.4)	41 (36.9)	59 (51.3)	0.006
Chronic pulmonary disease, *n* (%)	103 (22.9)	22 (19.8)	24 (21.2)	24 (21.6)	33 (28.7)	0.382
Charlson comorbidity index	5.0 ± 3.0	4.8 ± 3.2	5.2 ± 2.9	4.7 ± 3.1	5.0 ± 2.8	0.618
Vasoactive drugs day 1, *n* (%)	205 (45.6)	56 (50.5)	61 (54)	48 (43.2)	40 (34.8)	0.019
Blood culture, *n* (%)						0.003
Negative	210 (47.7)	38 (35.5)	61 (54)	57 (52.3)	54 (48.6)	
Staphylococcus spp	151 (34.3)	55 (51.4)	37 (32.7)	28 (25.7)	31 (27.9)	
Streptococcus spp	23 (5.2)	2 (1.9)	5 (4.4)	9 (8.3)	7 (6.3)	
Enterococcus spp	25 (5.7)	6 (5.6)	5 (4.4)	3 (2.8)	11 (9.9)	
HACEK	2 (0.5)	0 (0)	1 (0.9)	1 (0.9)	0 (0)	
Double infection	29 (6.6)	6 (5.6)	4 (3.5)	11 (10.1)	8 (7.2)	
Hemorrhagic events, *n* (%)	71 (15.8)	23 (20.7)	19 (16.8)	13 (11.7)	16 (13.9)	0.282
Thrombotic events, *n* (%)	221 (49.1)	76 (68.5)	50 (44.2)	50 (45)	45 (39.1)	<0.001

Abbreviations: MBP, mean blood pressure; PT, prothrombin time; WBC, white blood cell, PH, potential of hydrogen; HACEK, haemophilus, aggregatibacter, cardiobacterium, eikenella, kingella; spp, species.

### Relationship between platelet count and mortality

We performed four models (Model 1 was unadjusted. Model 2 adjusted for age, gender, race. Model 3 further adjusted for comorbidities congestive heart failure, renal disease, chronic pulmonary disease, and sepsis. Model 4: Adjust for age, sex, race, sepsis, congestive heart failure, chronic pulmonary disease, renal disease, creatinine, glucose, and CCI) to investigate the potential correlation between the platelet count and 28-day mortality in individuals with IE (Table 2). As a continuous variable, in the final adjusted model 4, per 10 unit increase in platelet count was associated with 2% decrease of mortality (HR = 0.98; 95% CI = 0.95–1; *p* = 0.04). When categorizing into platelet quartile, in the fully adjusted model 4, there was still a significant association between platelet count and 28-day mortality, the risk of 28-day mortality in Q4 group was significantly lower compared with that in Q1 group (Q4: HR = 0.53, 95%CI: 0.28–0.98, *p* = 0.042). The values of *p* for trend in all four models were both <0.05.

**Table 2:** Association between platelet count and 28-day mortality in different models.

	Model 1		Model 2		Model 3		Model 4	
	HR (95% CIs)	*P*	HR (95% CIs)	*P*	HR (95% CIs)	*P*	HR (95% CIs)	*P*
Platelet count per 10 (k/μL)	0.97 (0.95–0.99)	0.005	0.96 (0.94–0.99)	0.002	0.97 (0.94–0.99)	0.002	0.98 (0.95–1)	0.04
Platelet quartile, (k/μL)								
Q1(≤99)	1 (Ref)		1(Ref)		1(Ref)		1(Ref)	
Q2 (100–156)	0.54 (0.31–0.92)	0.024	0.45 (0.26–0.77)	0.004	0.46 (0.27–0.80)	0.006	0.53 (0.30–0.91)	0.023
Q3 (157–234)	0.42 (0.24–0.75)	0.004	0.40 (0.23–0.72)	0.002	0.41 (0.23–0.74)	0.003	0.44 (0.24–0.80)	0.007
Q4 (≥235)	0.42 (0.24–0.75)	0.003	0.36 (0.20–0.65)	0.001	0.37 (0.21–0.68)	0.001	0.53 (0.28–0.98)	0.042
*P* for trend		0.001		<0.001		0.001		0.015

Model 1: unadjusted. Model 2: adjust for age, sex, race. Model 3: adjust for age, sex, race, sepsis, congestive heart failure, chronic pulmonary disease, and renal disease. Model 4: Adjust for age, sex, race, sepsis, congestive heart failure, chronic pulmonary disease, renal disease, creatinine, glucose, and Charlson comorbidity index. Q, quartile; HR, hazard ratio; CI, confidence interval; Ref, reference.

### The nonlinearity relationship between platelet count and mortality

**Figure 2:**
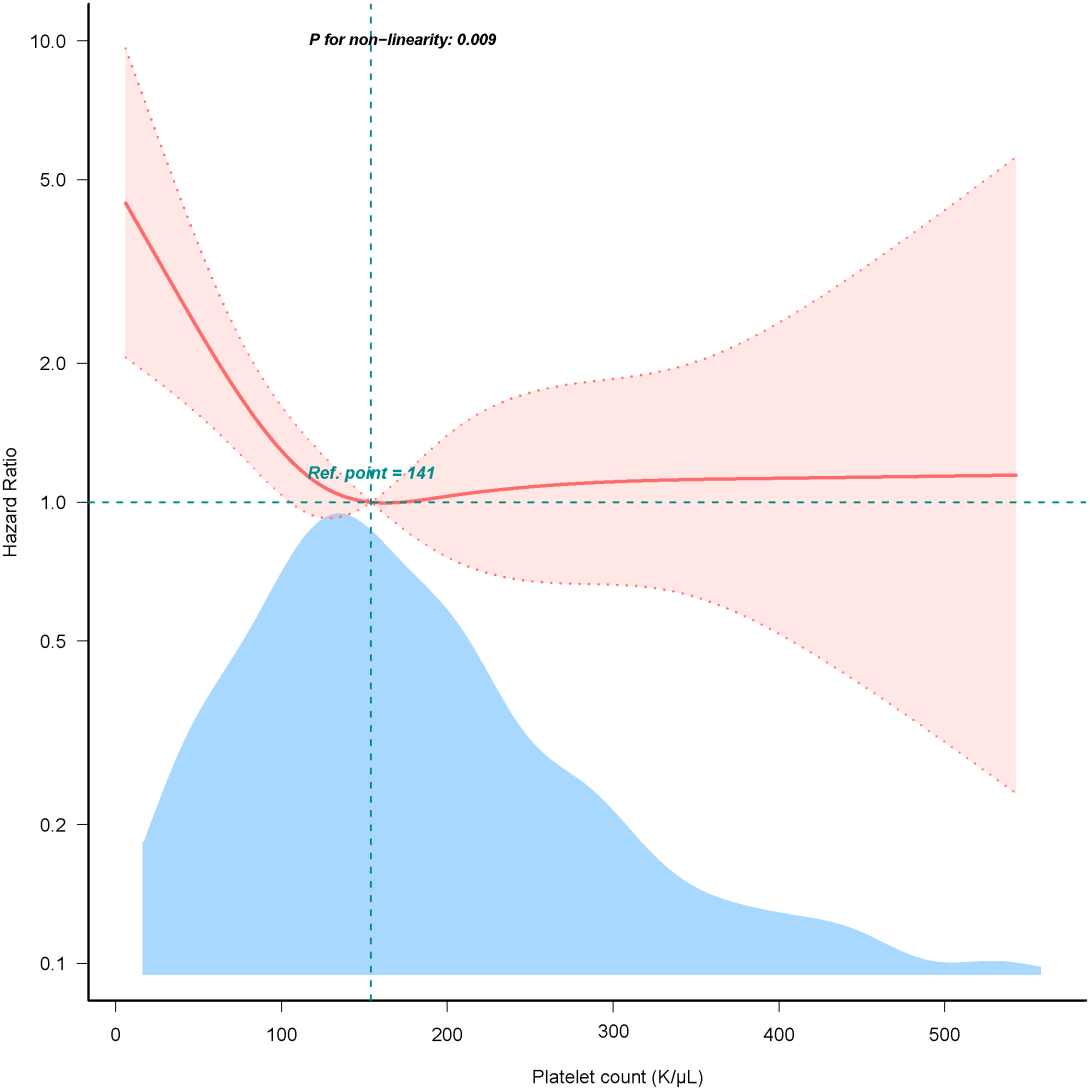
Dose-response relationship between platelet count with 28-day mortality. Adjusted for age, sex, race, sepsis, congestive heart failure, chronic pulmonary disease, renal disease, creatinine, glucose, and charlson comorbidity index. Only 99% of the data is shown.

We discovered that there was a non-linear connection between platelet count and in-hospital mortality using the multivariate Cox hazard regression model and smooth curve fitting (Figure 2). Since the non-linear test's *P*-value in our study was 0.005 (see Table 3), we employed a two-piecewise model to examine the relationship between platelet count and 28-day mortality. An inflection point was discovered at around 141 × K/µl (see Figure 2). On the left side of the inflection point, the hazard ratio was 0.990 (hazard ratio: 0.990, 95% CI: 0.982–0.997, *p* = 0.006). However, on the right side of the inflection point, the hazard ratio increased marginally to 1.0004 (HR: 1.0004, 95% CI: 0.997–1.004, *p* = 0.825). Notably, the association lacked statistical significance on the right side of the inflection point. It implies that until a platelet count of around 141 k/µl, the probability of death began to decline by 1% with every 1 k/µl platelet change.

**Table 3:** The non-linear relationship between platelet count and 28-day mortality.

Threshold of platelet count (k/µl)	HR	95% CI	*P*-value
<141	0.990	0.99 (0.982–0.997)	0.0062
≥141	1.0004	1.0004 (0.9967–1.0041)	0.8252
Non-linear test			0.005

Abbreviations: HR, hazard ratio; CI, confidence interval. HRs were adjusted for age, sex, race, sepsis, congestive heart failure, chronic pulmonary disease, renal disease, creatinine, glucose, and charlson comorbidity index. Only 99% of the data is displayed.

### Kaplan-Meier survival curve analysis

**Figure 3:**
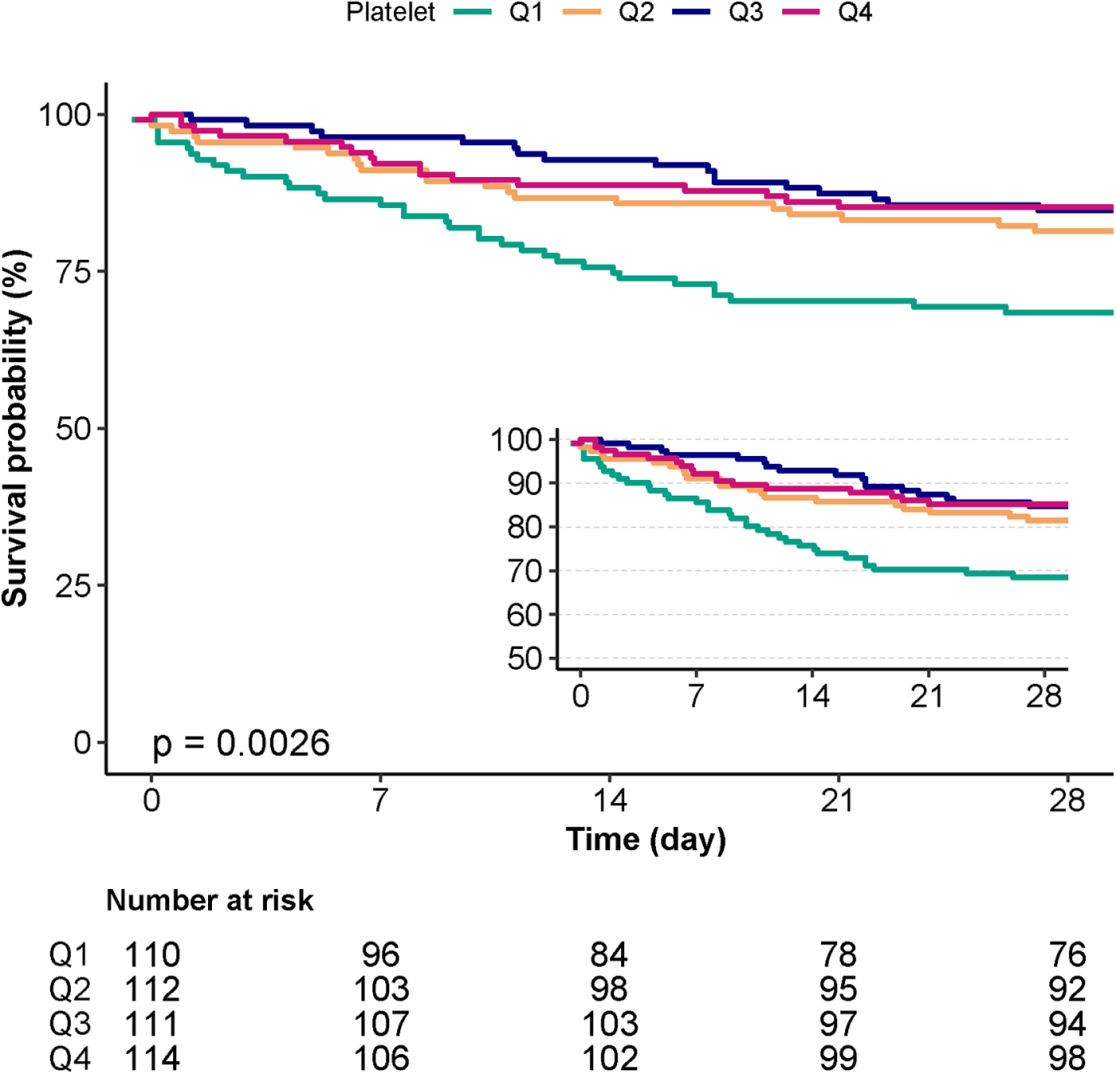
Kaplan-Meier survival curves for day 28 of patients with IE depending on the quartile of platelet count.

According to the Kaplan-Meier curve, the Q1 group's 28-day cumulative survival rates were lower than those of the other groups (*p* = 0.0026) (Figure 3).

### Subgroup analyses

**Figure 4:**
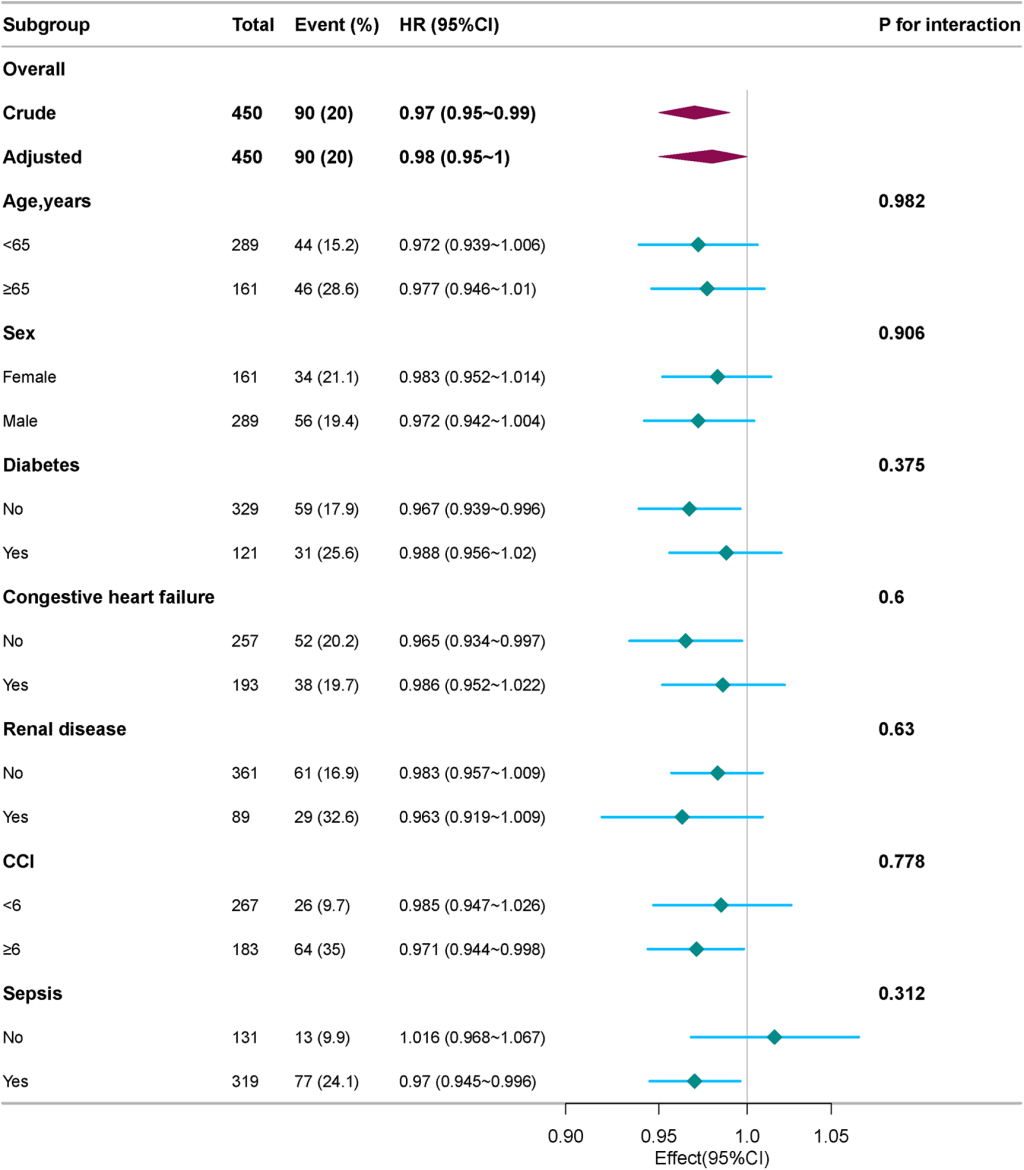
Subgroup and stratified analyses of the association between platelet count and 28-day mortality. Adjusted for age, sex, race, sepsis, congestive heart failure, chronic pulmonary disease, renal disease, creatinine, glucose, and charlson comorbidity index.

We conducted stratified and interaction analyses to evaluate the consistency of the association between platelet count and 28-day mortality in various subgroups including age (<65, or ≥65 years), sex, race, diabetes, congestive heart failure, renal disease, CCI (<6, or ≥6), and sepsis. Forrest plot demonstrated a persistent and independent association between platelet count and 28-day mortality in patients with IE (see Figure 4). In specific subgroups, no statistically significant associations were detected (*P* > 0.05).

### Sensitivity analysis

We conducted a sensitivity analysis using individuals with platelet counts above 141,000 as the reference group (normal). Furthermore, we subdivided individuals with platelet counts <141,000 into three categories: mild (PLT: 101–140 k/µl), moderate (50–100 k/µl), and severe thrombocytopenia (<50 k/µl). The results demonstrated that patients with severe thrombocytopenia faced a significantly higher risk of mortality compared to those with normal platelet counts (adjusted HR: 3.45, 95% CI: 1.93–6.15, *P* < 0.001) (Supplementary Table S1).

## Discussion

The results show a statistically significant decrease in the risk of death associated with greater platelet count. Interestingly, there are clear connections between platelet count and 28-day mortality on both sides of the inflection point, according to a threshold effect curve. An increase of 1 k/µl in platelet count is linked to a 1% reduction in mortality when platelet counts are below 141 k/µl. However, platelet counts above this threshold do not show a significant impact on mortality. This study, to the best of our knowledge, is the first to investigate the association between platelet count and mortality in critically ill patients with infective endocarditis (IE). There's been more focus on the relationship between platelet count and unfavorable outcomes in critically sick individuals. Jonsson et al. (25) found a correlation between thrombocytopenia and higher mortality in a cohort study which included 215,098 ICU patients. In a cohort research involving 8,025 stroke patients in the ICU, Wang et al. (14) found a significant negative correlation between platelet count and 30-day in-hospital mortality. In addition, Zhou et al. (15) found an inverse relationship between the platelet count and 30-day in-hospital mortality in 22,262 ICU patients suffering from acute respiratory failure. Previous research on sepsis (11), covid-19 (12, 13) consistently indicated a correlation between lower platelet counts and increased mortality rates in critically ill patients. These studies highlight the platelet count as an important prognostic factor for patients in the ICU who are critically ill. Our study's outcome is comparable to those of the other research mentioned. Liu et al. (26) reported mean platelet volume/platelet count ratio (MPR) was associated with all-cause mortality in patients with infective endocarditis from a Chinese hospital. Another Chinese team, Wang et al. (27) displayed that preoperative platelet count has a significant association with 1-year all-cause mortality and adverse postoperative outcomes in patients with IE. These studies highlight the platelet count as an important prognostic factor for patients in the ICU and patients with IE.

Building on prior studies, we suggest that thrombocytopenia and elevated 28-day mortality may have a shared underlying mechanism. Firstly, an excess of reactive oxygen species (ROS) decreases megakaryocytes proliferation and platelet formation (28). Meanwhile, inflammatory factors such as TNF-α, IFN-α and IFN-r suppress platelet production (28, 29). Secondly, platelets are vital for hemostasis and the coagulation process (30). Thrombocytopenia increases the risk of primary or secondary hemorrhage in vital organs, thereby lowering overall survival rates, which is especially concerning in critical ICU settings. Thrombocytopenia acts as an indirect marker of greater illness severity (31–33). Patients with lower platelet counts frequently need more intensive medical interventions, such as the use of vasoactive drugs, renal replacement therapy, and mechanical ventilation. Moreover, platelets are recognized as the initial agents of innate immunity, interacting through a variety of surface receptors with pathogens, such as bacteria and viruses (34, 35). In addition to functioning as immune cells, platelets communicate with neutrophils, monocytes, dendritic cells, and lymphocytes (36). These variables imply that individuals with lower admission platelet count may have weakened immune systems, which would increase their mortality risk. The observed negative connection between platelet count and 28-day mortality on the left side of this study's inflection point is probably caused by these reasons. On the other hand, this association loses statistical significance on the right side of the inflection point, maybe as a result of deteriorating patient circumstances and more confounding variables. Thus, it is crucial, according to the authors, to maintain a proper platelet count.

Our study has several noteworthy strengths. Firstly, it is among the first to investigate the role of the platelet count in patients with IE in the ICU. Secondly, we utilized smooth curve fitting to examine nonlinear relationships between the platelet count and outcome. Additionally, to account for potential confounding variables, we conducted cox regression analysis, incorporating multiple models, and performed subgroup analyses with appropriate categorizations.

However, our study also has several limitations, commonly found in retrospective studies. Firstly, the observational study design precludes establishing causality. Secondly, the use of administrative data introduces the potential for misclassification bias, as diagnoses are often based on ICD codes, which may be subject to errors in coding or incomplete documentation. Thirdly, the study did not specifically aim to determine the reasons for the decrease in platelet count in critically ill patients, thus preventing us from inferring the pathophysiological mechanisms underlying the decrease in platelet count and its association with increased patient mortality. While infection-related disseminated intravascular coagulation has been reported as the most common cause of decrease or increase in platelet count, other factors such as liver disease, hematologic disorders, massive transfusions, drug-induced thrombocytopenia, and immune-mediated thrombocytopenia may also play a role. Therefore, regardless of the specific mechanism, the decrease in platelet count may be a strong indicator for evaluating patient prognosis. This study should serve as the basis for well-designed future studies to assess the impact of decreased platelet count on mortality and causality.

## Conclusion

A nonlinear association between platelet count and 28-day mortality was observed in critically ill patients with infective endocarditis. The optimal platelet count associated with the lowest risk of 28-day mortality was 141 k/µl.

## Data Availability

The raw data supporting the conclusions of this article will be made available by the authors, without undue reservation.
