# Supplementary material for: Non-linear relationship between platelet count and 28-day mortality in critically ill patients with infective endocarditis: a retrospective cohort study from MIMIC IV database
Source: Front Cardiovasc Med. 2024 Nov 29;11:1458238. doi: 10.3389/fcvm.2024.1458238 (PMC11638226; doi:10.3389/fcvm.2024.1458238)
Supplement: Supplementary file 1 [file Table1.docx]

Table S1 Association Between Platelet Count and 28-day Mortality in Different Platelet Group

| Platelet ((k/uL)) | n.total | n.event_% | Crude model | | Adjusted model | |
| --- | --- | --- | --- | --- | --- | --- |
|  |  |  | HR (95%CI) | *P* value | HR (95%CI) | *P* value |
| Normal (≥141) | 257 | 38 (14.8) | 1(Ref) |  | 1(Ref) |  |
| Severe (<50) | 49 | 20 (40.8) | 3.52 (2.05~6.05) | <0.001 | 3.45 (1.93~6.15) | <0.001 |
| Moderate (50-100) | 62 | 15 (24.2) | 1.71 (0.94~3.11) | 0.078 | 1.46 (0.79~2.71) | 0.228 |
| Mild (101-140) | 82 | 17 (20.7) | 1.48 (0.83~2.62) | 0.182 | 1.25 (0.7~2.23) | 0.458 |
| *P* for trend | 450 | 90 (20) |  | <0.001 |  | <0.001 |

Adjusted for age, sex, race, sepsis, congestive heart failure, chronic pulmonary disease, and renal disease.
